# Carpal Tunnel Syndrome and Diabetes—A Comprehensive Review

**DOI:** 10.3390/jcm11061674

**Published:** 2022-03-17

**Authors:** Malin Zimmerman, Anders Gottsäter, Lars B. Dahlin

**Affiliations:** 1 Department of Hand Surgery, Skåne University Hospital, Lund University, 205 02 Malmö, Sweden; lars.dahlin@med.lu.se; 2 Department of Translational Medicine—Hand Surgery, Lund University, 205 02 Malmö, Sweden; 3 Department of Orthopaedic Surgery, Helsingborg Hospital, 251 87 Helsingborg, Sweden; 4 Department of Medicine, Skåne University Hospital, 205 02 Malmö, Sweden; anders.gottsater@med.lu.se; 5 Department of Clinical Sciences Malmö, Lund University, 205 02 Malmö, Sweden; 6 Department of Biomedical and Clinical Sciences, Linköping University, 581 83 Linköping, Sweden

**Keywords:** carpal tunnel syndrome, diabetic neuropathy, diabetes

## Abstract

Carpal tunnel syndrome (CTS) is the most common compression neuropathy in the general population and is frequently encountered among individuals with type 1 and 2 diabetes. The reason(s) why a peripheral nerve trunk in individuals with diabetes is more susceptible to nerve compression is still not completely clarified, but both biochemical and structural changes in the peripheral nerve are probably implicated. In particular, individuals with neuropathy, irrespective of aetiology, have a higher risk of peripheral nerve compression disorders, as reflected among individuals with diabetic neuropathy. Diagnosis of CTS in individuals with diabetes should be carefully evaluated; detailed case history, thorough clinical examination, and electrophysiological examination is recommended. Individuals with diabetes and CTS benefit from surgery to the same extent as otherwise healthy individuals with CTS. In the present review, we describe pathophysiological aspects of the nerve compression disorder CTS in relation to diabetes, current data contributing to the explanation of the increased risk for CTS in individuals with diabetes, as well as diagnostic methods, treatment options, and prognosis of CTS in diabetes.

## 1. Introduction

Nerve compression disorders are common among the general population, and the most frequently encountered lesions are carpal tunnel syndrome (CTS) and ulnar nerve entrapment at the elbow (UNE) [1]. Even if both conditions are considered as nerve compression disorders, they have substantially different characteristics, regarding the socioeconomic background of affected individuals as well as the individual nerve’s susceptibility and reaction to trauma; factors that both impact outcome of surgical procedures [2,3]. The prevalence of CTS is 2.7% (depending on its definition) and the yearly incidences are 428 in women and 182 in men per 100,000 adults in Sweden [4,5], but figures may differ between both regions and countries [6,7,8]. In addition, the annual incidences of CTS surgery are higher in Sweden and the United States compared to, e.g., in the United Kingdom [1,5]. The aetiology behind CTS is multifactorial, and both intrinsic and extrinsic factors; i.e., factors related to both the peripheral nerve and the surroundings of the nerve trunk have to be considered. Frequent causes of CTS are endocrine disorders, like hypothyroidism, pregnancy, menopause, obesity, diabetes, Hand Arm Vibration Syndrome (HAVS), rheumatoid arthritis, traumatic injuries, such as fractures and dislocations of the distal radius and carpal bones, and repetitive motions of the wrist. However, in the majority of CTS cases, no specific cause can be identified, and the condition is considered as idiopathic. Recently, genome-wide association studies (GWAS) have reported 16 susceptibility loci for CTS [9], and as the variants in those genes are implicated in both growth (i.e., anthropometric measurements) and enrichment of extracellular matrix architecture, the genetic risks are related to the environment of the carpal tunnel as well as to the vulnerability of the median nerve fibres to compression. CTS is also considered to be a part of the diabetic hand, which includes not only limited joint mobility, Dupuytren’s disease with contracture, and flexor tenosynovitis (i.e., trigger finger) [10,11], but also ulnar nerve compression at the elbow (UNE) [12]. Diabetes increases the risk of compression neuro-pathies [13,14], and a prominent feature may be inherent factors in the peripheral nerve trunk, comparable to HAVS [15,16,17]. This phenomenon is included in the double crush theory; a nerve already affected by some pathology is more susceptible to compression [15].

Here, we present an overview of the pathophysiology and vulnerability of the median nerve in CTS, with relevant diagnostic procedures, treatment options, and outcome of surgery in individuals with CTS and diabetes.

## 2. Neuropathy in Diabetes

The prevalence of diabetic neuropathy is estimated to be 30–50% in individuals with diabetes [18,19,20], and it increases with disease duration. Importantly, diabetic neuropathy may be present already at the time of diagnosis [21,22], where diabetic men are more prone than diabetic women to develop neuropathy [23]. The most common type of diabetic neuropathy is distal symmetric polyneuropathy, a major cause of diabetic foot complications [21]. Other neuropathy types in diabetes are autonomic neuropathy and mononeuropathies, including compression neuropathies [21].

**Figure 1:**
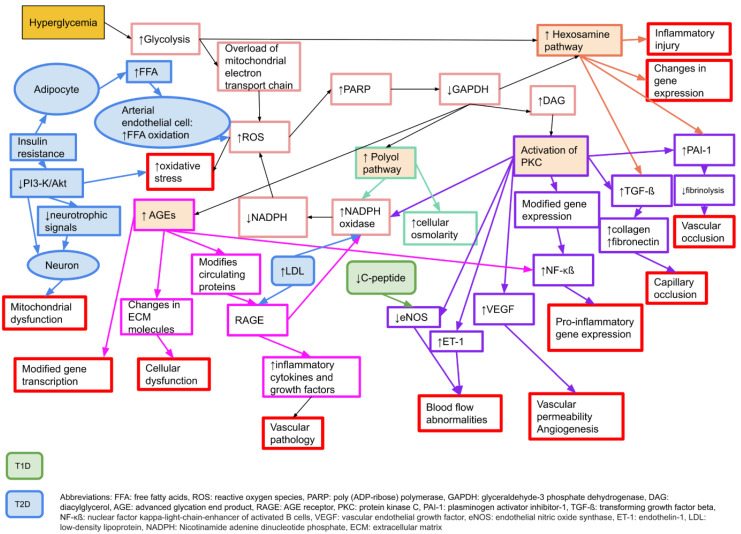
Molecular mechanisms behind microvascular complications in diabetes that may affect neurons, Schwann cells, and vascular endothelial cells, causing neuropathy or nerve dysfunction. Adapted from Zimmerman 2018 [26] with permission. T1D: type 1 diabetes, T2D: type 2 diabetes.

There are several proposed mechanisms behind the development of neuropathy in diabetes. The main causal factor is considered to be hyperglycaemia [24], which leads to an increased oxidative stress and an increase in free radicals [25]. Pathophysiologically, there are four key elements behind the hyperglycaemic damage to peripheral nerves; increased activity in the polyol pathway, activation of protein kinase C (PKC), production of advanced glycation end products (AGE), and an increased activity of the hexosamine pathway [24]. For details, please see Figure 1.

**Figure 2:**
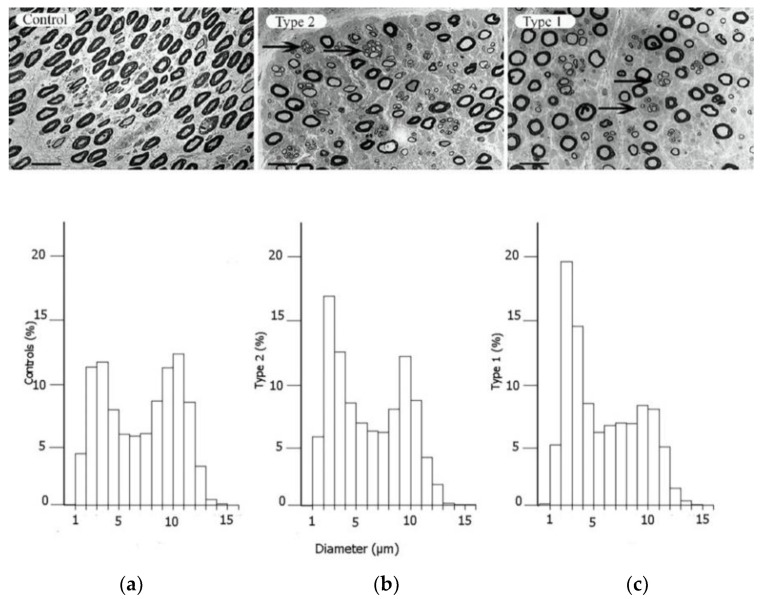
Electron micrographs of the posterior interosseous nerve, with diagram of size distribution of myelinated nerve fibres, from patients with CTS, where the individuals are healthy (**a**), have type 2 diabetes (**b**) or type 1 diabetes (**c**). The arrows in the upper panels indicate regenerative clusters. In the diagram on the lower panels, the size distribution of myelinated nerve fibres is based on the micrographs from the upper panels, indicating a redistribution of nerve fibres. Scale bar = 20 µm. Reproduced by kind permission by Osman et al., Diabetologia 2015 [39].

Alterations in axonal transport have also been described in experimental studies of diabetes [27,28]. These changes result in reduced axon calibre, segmental demyelination, and loss of myelinated nerve fibres [29] (Figure 2). Both micro- and macrovascular alterations in diabetes add additional stress on peripheral nerves. However, the mechanisms behind diabetic neuropathy differ between type 1 and type 2 diabetes [18,30,31]. In type 1 diabetes, intensive glucose control protects against neuropathy development [30,31], and insulin deficiency might contribute to neuropathy since insulin has a neurotrophic effect [32]. The loss of C-peptide in type 1 diabetes might also contribute to hypoxia by lowering eNOS [33]. In contrast, in type 2 diabetes, both hyperlipidaemia and insulin resistance may play a part in the development of neuropathy [32,34,35,36]. There is also evidence that glucose control may have a modest effect on lowering neuropathy complications in type 2 diabetes [37,38].

## 3. The Increased Susceptibility to Nerve Compression in Diabetes

A peripheral nerve trunk is a delicate structure, where the various components respond to an external trauma in different ways (for a classical review, see Sunderland 1978 [40]). The axons, with their associated Schwann cells, are enclosed by a basement membrane, and the myelinated and unmyelinated nerve fibres are assembled in bundles surrounded by a strong connective tissue layer with flattened cells (perineurium), providing both chemical and mechanical protection [40,41]. The connective tissue component inside the perineurial sheath is called the endoneurium. The bundles of nerve fibres with the perineurium (i.e., fascicles) are embedded in loose connective tissue components—epineurium. The nerve trunk is segmentally provided by small blood vessels that branch into the different connective tissue compartments, where the endoneurial blood vessels, mainly capillaries, are strongly resistant to trauma [42]. In contrast, the epineurial blood vessels are sensitive to trauma with a risk of formation of epineurial oedema that may later form into fibrosis. The number of nerve fibres with a larger diameter, i.e., the myelinated fibres, are reduced, particularly in type 1 diabetes, with a resulting bimodal distribution of nerve fibres (Figure 2); i.e., a higher number of unmyelinated nerve fibres [39]. Myelinated nerve fibres are also more sensitive to nerve compression trauma than un-myelinated nerve fibres [43].

The intra-axonal communication system consists of a delicate system of anterograde and retrograde transport of various substances, such as structural, metabolic, and growth-related proteins, known as axonal transport, which is of utmost relevance in health and disease [44]. Axonal transport can not only be disturbed in diabetes with the development of neuropathy, but can be inhibited by applied nerve compression [27,45]. In experimental studies using diabetic rats, local compression of a nerve causes an increased inhibition of axonally transported proteins compared to in healthy rats [45]; the concept is conceivable that a nerve is more susceptible to compression when the peripheral nervous system is affected by a generalised disease, such as diabetes [15]. In this context, one has also to consider all related disturbances in diabetes, such as those occurring in the red blood corpuscles, the extra- and intraneural blood vessels, and in the connective tissue components whether in the nerve trunk or in the surroundings as in the carpal ligament, e.g., with glycosylation of collagen. The glycosylation of collagen leads to an increase in advanced glycation end products (AGE), causing cross-linking of collagen fibres in the transverse carpal ligament, resulting in increased stiffness and contributing to space limitation in the carpal tunnel [46]. Nerve oedema, originating from an increased vascular permeability and angiogenesis, due to upregulation of vascular endothelial growth factor (VEGF) in diabetes, may also contribute to the increased susceptibility to compression trauma [47]; a mechanism that has been related to increased endoneurial pressure with a risk of jeo-pardised blood supply to the nerve fibres in the fascicles [48]. Nerve oedema has been demonstrated in ultrasound studies, showing that the median nerve cross-sectional area is enlarged in diabetes [49]. Upregulation of VEGF and its receptors, but not of the hypoxia-inducible factor 1α (HIF1α), has been demonstrated in biopsies of the posterior interosseus nerve (PIN) from patients with CTS and diabetes [50]. The number of myelinated nerve fibres has been analysed in such biopsies of the posterior interosseous nerve, indicating that otherwise healthy subjects with CTS have a lower density of myelinated nerve fibres than those without CTS [16]. Interestingly, patients with diabetes and CTS have an even lower density of such myelinated nerve fibres, which may explain the increased susceptibility [16]. In accordance, subjects with HAVS also have structural changes in upper extremity nerves [51], resulting in a higher risk for additional CTS [17]. To conclude, diabetes may confer an increased susceptibility to the peripheral nerve, where the pathophysiological mechanisms are complex and involve both biochemical and structural alterations in the nerve.

## 4. Symptoms and Clinical Signs of CTS

In CTS, symptoms, clinical findings, and electrophysiology results depend on the magnitude, nature, and duration of the compression trauma. In individuals with diabetes, early signs of CTS may be mistaken for diabetic neuropathy [52]. Basically, one may relate the pathophysiological events in the nerve to the experienced symptomatology, irrespective of whether the affected individual has diabetes or not. Initially, due to the distur-bances in intraneural microcirculation and possible dynamic ischemia by a slight compression trauma, paraesthesia, and numbness are induced in the median nerve innervated sensory area of the affected hand with worse symptoms during the night [53]. This might be detected as a metabolic conduction block on electrophysiology testing. As the compression worsens, endoneurial oedema may form, causing increased endoneurial pressure with further microcirculatory disturbances [54] and more constant symptoms. Eventually, the compression trauma at this stage leads to demyelination [55,56] and at a later stage, even axonal degeneration [57] with end-stage symptoms, such as anaesthesia and thenar atrophy [56]. The focal demyelination can be detected on the electrophysiology examination as an increased latency with a decrease in nerve conduction velocity [58], while axonal degeneration is reflected in a reduced amplitude [59,60]. Patients with diabetes and CTS are twice as likely to present with advanced disease as measured by electrophysiology than those with CTS without concomitant diabetes [61]. After surgery, with the release of the carpal ligament, recovery of patient symptoms also depends on preoperative severity. The microcirculatory disturbances recover quickly, while the structural changes may disappear slowly or incompletely. Remyelination follows the demyelination process, but remyelinated segments have thinner myelin [62] with shorter internodal distances, observed as a permanently reduced conduction velocity. Recovery, requiring axonal regeneration in the nerve, may take longer and may be incomplete, with a reduced amplitude on the electrophysiological examination.

## 5. CTS and Type 1 and Type 2 Diabetes

In a recent study from the UK of 401,656 individuals, including 24,558 with diabetes, the odds ratio (OR) for CTS in diabetes was 2.31 (95% CI 2.17–2.46) [63]. Similar results were presented in a Swedish cohort study of 30,466 individuals showing a hazard ratio (HR) of 2.10 (95% CI 1.65–2.70) [14], and the pooled OR was 1.69 (1.45–1.96) in one large review controlling for confounders [64]. A meta-analysis indicated that associations with CTS were the same in type 1 and 2 diabetes [64], but more current research has confirmed that CTS is more common in type 1 patients (9). Incidence rates for CTS are reported to be 95.5/10,000 person-years for women and 58.1/10,000 person-years for men with type 1 diabetes, and 52.1/10,000 person-years for women and 31.6/10,000 person-years for men with type 2 diabetes [12] (Table 1). The higher incidence rates in type 1 diabetes may be attributed to the presence of neuropathy, which can be detected as alterations in the distribution of nerve fibres of different sizes in the posterior interosseous nerve (PIN) with more autophagy-related ultrastructures [39].

## 6. Sex Differences in CTS and Diabetes

Diabetic neuropathy might affect CTS presentation differently in men and women. Men generally present with worse electrophysiology results [65,66], which might be attributable to the higher prevalence of diabetes among men with CTS. Diabetic neuropathy also develops earlier and to a greater extent in men than in women [23,67]. In skin biopsies at wrist level, men have lower intraepidermal nerve fibre density (IENFD) than women, indicating that there might be less spare capacity in men, making male nerves more susceptible to compression trauma, such as CTS [68]. This does not, however, seem to translate to symptom presentation, as women with CTS and diabetic neuropathy experience more symptoms than men [69].

## 7. Value of Electrophysiology in CTS and Diabetes

Routines for preoperative electrophysiology testing to diagnose nerve compression disorders differ between countries. In Sweden, electrophysiology is not mandatory, but is often used to strengthen the diagnosis of CTS. In previous studies, 70% of all patients with CTS had undergone electrophysiology testing before surgery [70]. A general agreement seems to be that electrophysiology is not necessary in uncomplicated cases, but can aid in differential diagnosis and in prognosticating surgical outcomes [71], which might be crucial in individuals with diabetes.

In individuals with both diabetic polyneuropathy and CTS, the use of electrophysiology has been debated [72], as diabetic polyneuropathy might obscure the electrophysiological findings of CTS [72,73], and electrophysiology does not always reveal a predominantly small-fibre neuropathy [32]. It may, however, be of value to generously admit individuals with diabetes and CTS for electrophysiology testing to diagnose potential polyneuropathy, enabling a better prognostication regarding surgical outcomes. In this setting, it might also be relevant to perform electrodiagnostic testing also on the lower extremities to evaluate the degree of diabetic neuropathy, as a potential differential diagnosis to CTS. However, if the individual has evident CTS, electrophysiology testing should not delay surgical treatment.

## 8. Treatment Options

There is strong evidence that the surgical release of the carpal ligament provides better symptom relief in CTS than conservative treatment options, including splinting, corticosteroid injection, and oral NSAIDs during follow-up, up to 18 months [74,75,76]. About two-thirds of individuals with CTS are treated surgically [77]. Historically, among some physicians, there has been a cautious approach to carpal tunnel release in CTS in individuals with diabetes, based on the notion that they may not benefit from surgery to the same extent as individuals without diabetes [78,79]. As a result of this, current research indicates that persons with long-term diabetes and CTS might still be undertreated [80].

## 9. Outcome of Surgery

The surgical outcome for CTS is difficult to compare between studies, since there is a lack of consensus on how to measure and evaluate nerve function and patient-related outcomes. In one prospective series, with extensive outcome measurements using monofilament, 2-point discrimination (2PD), the strength of the abductor pollicis brevis muscle, grip- and pinch strength, pillar pain, and a VAS-questionnaire up to five years postoperatively, individuals with diabetes improved to the same extent as those without diabetes; the only observed difference at one year being remaining cold sensitivity in diabetes [81,82]. In the same cohort, electrophysiological measurements improved over five years in both individuals with and without diabetes and were not influenced by the presence of neuropathy [59,60]. Another prospective study, using QuickDASH, found that individuals with CTS and diabetes had poorer functional scores at 12 months postoperatively than those without diabetes (mean difference 7.5 points in the QuickDASH), but it is doubtful whether this difference was of clinical significance [83]. One retrospective study, comparing individuals with type 2 diabetes to those without diabetes, reported higher frequencies of night time pain, weakness, and paraesthesia in those with type 2 diabetes both pre- and postoperatively, and a higher frequency of postoperative numbness among individuals with diabetes following open carpal tunnel release (OCTR) [84]. Another retrospective study, using the Boston Carpal Tunnel Questionnaire, found no differences in surgical outcome at six months postoperatively between individuals with and without diabetes [85]. Two other prospective studies using the Boston Carpal Tunnel Questionnaire reported similar results; however, individuals with diabetes took longer times to improve [86] and had more symptoms at a 10-year follow-up [87]. In one prospective study on the resolution of daytime numbness, however, diabetes did not affect surgical results [88]. Another study, using a symptom score, reported substantial improvement in individuals with non-insulin-dependent diabetes, but this group still had more residual symptoms compared to individuals without diabetes [79].

Concomitant diabetic polyneuropathy has been associated with more residual symptoms, measured by QuickDASH at 12 months, following OCTR (i.e., median postoperative QuickDASH score of 61 compared to 20 in diabetic individuals without polyneuropathy) [89]. Worse diabetic control, reflected by higher preoperative HbA1c levels, has also been associated with higher QuickDASH scores at 12 months after OCTR. In the same study, those with diabetic retinopathy recovered slower than individuals with diabetes without retinopathy [90]. One recent meta-analysis concluded that there were no differences in improvement of patient-reported outcomes following carpal tunnel release between individuals with and without diabetes. Among the electrophysiology results, the only variable that showed less improvement in individuals with diabetes was the sensory conduction velocity [91], possibly due to pre-existing diabetic neuropathy. There is also data indicating that individuals with prediabetes have a worse surgery outcome than individuals without diabetes [90]. In theory, CTS could be the first symptom of diabetes, but screening individuals with CTS for diabetes has not been proven to be cost-effective [92]. Please see Table 2 for an overview of studies evaluating outcomes after OCTR in diabetes.

Individuals with diabetes also have a higher risk of surgical wound infection following OCTR [93], although infection rates are generally low [94]. Although higher preoperative HbA1c levels are associated with a higher frequency of surgical site infections [95], routine screening with HbA1c for the purpose of lowering surgical complications is not clinically valuable [96]. In all, there is substantial evidence that individuals with diabetes and CTS benefit from surgery.

**Table 2:** Overview of studies evaluating outcome after open carpal tunnel release in individuals’ CTS and with and without diabetes.

Author, Year	Study Design	N of Individuals (Hands)	Diabetes	Type of Diabetes	Neuropathy	Outcome Measure	Follow-Up Time	Results, Diabetes vs. No Diabetes
Haupt 1993 [78]	Prospective	60 (86)	10/60 (17%)	Not reported	Not reported	Motor function, sensory deficit, trophic changes, neurography and electro-myography	5.5 years	Marginally less pain relief in individuals with diabetes
al-Qattan 1994 [97]	Retrospective	15 (20)	15/15 (100%)	Not reported	15/15	Grading: excellent/good/poor	18 months	5 hands had poor improvement—all of these had normal/mild neurography pre-op
Choi 1998 [98]	Retrospective	154 (294)	19/154 (12%)	Not reported	3 (1.9%)	Symptom resolution (poor-excellent)	12 months	No difference
Ozkul 2002 [79]	Prospective	47 (60)	22/47 (47%)	T2D	Excluded	PROM: global symptom score, neurography	12 months	Better PROMs and neurography recovery in individuals without diabetes
Mondelli 2004 [99]	Prospective case series	96 (96)	24/96 (25%)	T1D: 19 T2D: 5	6/24 (25%)	BCTQ	6 months	No difference
Thomsen 2009 [81]	Prospective	66 (66)	35/66 (53%)	T1D: 15 T2D: 20	14/35 (40%)	Monofilament, 2PD, APB strength, grip strength, key pinch, lateral pinch, pillar pain, postoperative questionnaire (VAS questions)	52 weeks	Individuals with diabetes had the same beneficial outcome after carpal tunnel release as non-diabetes individuals
Thomsen 2010 [59]	Prospective	66 (66)	35/66 (53%)	T1D: 15 T2D: 20	14/35 (40%)	Electrophysiology testing	12 months	Electrophysiology improved as much in individuals with as without diabetes
Jenkins 2012 [83]	Prospective	1564 (1564)	176/1564 (11.3%)	Not reported	Not reported	QuickDASH	12 months	Poorer functional scores after 12 months in individuals with diabetes, but doubtful whether of clinical significance
Isik 2013 [84]	Retrospective case-control	74 (99)	36/74 (49%)	T2D	none	PROM questions on symptoms	12 months	Worse post-op symptoms in individuals with diabetes
Zyluk 2013 [85]	Retrospective	386 (386)	41/386 (11%)	T1D: 11 T2D: 30	None	BCTQ	6 months	Clinical benefit: no difference. DM individuals had weaker grip strength and poorer perception of touch
Ebrahimzadeh 2013 [100]	Retrospective	74 (74)	35/74 (47%)	T1D: 14 T2D: 21	Not reported	WHOQOL-BREEF; MHQ	3 months	Worse results in individuals with diabetes, MHQ-scores better in T2D than T1D
Cagle 2014 [86]	Prospective	826 (950)	90/950 (10%)	Not reported	20/950 (2%)	BCTQ	12 weeks	Individuals with diabetes improved but took longer
Gulabi 2014 [87]	Prospective	69 (69)	27/69 (39%)	T1D: 18 T2D: 9	Not reported	BCTQ	10 years	Individuals with diabetes worse at the 10 years follow-up. No difference at 6 m.
Thomsen 2014 [82]	Prospective	66 (66)	35/66 (53%)	T1D: 15 T2D: 20	14/35 (40%)	BCTQ, monofilament, 2PD, APB strength, grip strength, key pinch, lateral pinch, pillar pain, VAS questions	5 years	Excellent long-term improvement in individuals with diabetes
Yucel 2015 [101]	Retrospective	83 (101)	35/83 (42%)	Not reported	Not reported	VAS-questions, BCTQ, monofilament, grip and pinch strength	Not specified	Individuals with diabetes had more symptoms in BCTQ
Zimmerman 2016 [89]	Retrospective	493 (531)	76/531 (14%)	T1D: 18 T2D: 58	18/76	QuickDASH	12 months	Same improvement, but more persistent symptoms in individuals with diabetes and polyneuropathy
Thomsen 2017 [60]	Prospective	57 (57)	27/57 (47%)	T1D: 13 T2D: 14	10/27 (37%)	Electrophysiology parameters	5 years	Long-term electrophysio-logy improvement was seen in both diabetes and non-diabetes individuals
Watchmaker 2017 [88]	Prospective	1031 (1037)	133/1031 (13%)	Not reported	Not reported	Symptom survey	6 months	Individuals with diabetes had the same symptom resolution
Zhang 2018 [102]	Retrospective	904 (1144)	Not reported	Not reported	Not reported	Secondary surgery	60 months	DM associated with greater risk of secondary surgery
Zimmerman 2019 [90]	Retrospective	9049 (10,770)	1508/9049 (17%)	T1D: 335 T2D: 1150	Not reported	QuickDASH	12 months	Individuals with diabetes benefitted from surgery, but not to same extent as patients without diabetes

APB: adductor pollicis brevis muscle, BCTQ: Boston Carpal Tunnel Questionnaire, DM: diabetes mellitus, PROM: Patient-reported outcome measure, QuickDASH: short version of disabilities of arm, shoulder and hand, T1D: type 1 diabetes, T2D: type 2 diabetes, 2PD: two-point discrimination, VAS: visual analogue scale.

## 10. Controversies in Nerve Compression and Diabetes

Current data clearly states that individuals with CTS and diabetes benefit from surgery when the diagnosis of CTS is obvious [59,60,78,79,81,82,83,84,85,86,87,88,89,90,97,98,99,101]. This conclusion should not be confused with the discussions about the surgical release of nerves in the lower extremity. It has been suggested that nerves in the lower extremity of individuals with diabetes and sensorimotor polyneuropathy should be decompressed as a preventive procedure against “superimposed nerve entrapment”, with the intention to prevent diabetic foot ulcers [103,104,105]. The indications for such procedures have been questioned [106], but recent opinions have been raised that the scepticism concerning such a procedure should be reassessed [107]. To solve the question, standard definitions and outcome measures are used in prospective randomised controlled trials to determine the usefulness of such interventions [108,109]. However, there are no data to support that peripheral nerves in the upper extremity, such as the median nerve at carpal tunnel or the ulnar nerve at the elbow, should be surgically released on broader indications than presently performed.

## 11. Future Perspectives—The Diabetic Nerve

**Figure 3:**
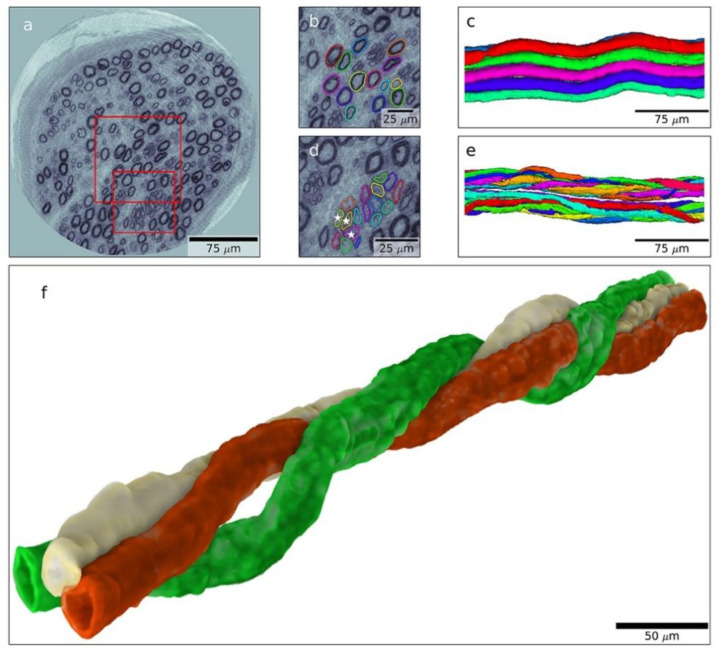
Nanotomogram with 3D images of a posterior interosseous nerve biopsy from an individual with type 1 diabetes. A tomographic slice (**a**) with an enlarged area (**b**) from which a 3D image is created with normal myelinated nerve fibres (**c**). Enlarged area (**d**) with a regenerative cluster, i.e., regenerating nerve fibres (**e**), and with details of such a regenerative cluster (**f**), showing spiral-shaped nerve fibres that have regenerated. Length of bar indicated in the figure. Reproduced by kind permission from Dahlin et al., Scientific Reports 2020 [110].

In order to improve the care of the diabetic hand, physicians and health care staff should ask simple questions and perform modest clinical tests for screening of diabetic hand-related diseases, such as CTS and UNE at the elbow. This could potentially lead to faster diagnosis and treatment. Hopefully, future studies will also shed more light on the pathophysiology of diabetic neuropathy, enabling the use of novel techniques. Recently, X-ray phase-contrast holographic nanotomography has been used to reveal the three-dimensional architecture of the nerve fibres in a human nerve (Figure 3). Interestingly, details of normal as well as degenerating and regenerating nerve fibres have been visualised, particularly the architecture of regenerative clusters in nerve biopsies from diabetic subjects [110] (Figure 3). This novel technique can be combined with mass spectrometry to analyse the proteomics in the nerve biopsies [111]. Identification of individuals at risk for the development of neuropathy, as well as CTS or UNE at the elbow, is crucial, with the intention of appropriately timed diagnosis and treatment [9]. Finally, national registers, in which diagnosis and outcomes of surgery of thousands of patients are assembled [90], constitute an additional step towards further refined treatment strategies in neuropathy and CTS.

## 12. Conclusions

Multiple mechanisms, including both biochemical and structural factors, contribute to the susceptibility of peripheral nerves to compression in diabetes, where CTS is more common among individuals with diabetes. CTS is more common in type 1 diabetes than in type 2 diabetes. A meticulous case history as well as a thorough clinical and electrophysiological examination are recommended to support the diagnosis and reveal neuro-pathy in individuals with diabetes and suspected CTS. Individuals with diabetes with CTS benefit from surgical treatment to the same extent as individuals with CTS but without diabetes. Symptom resolution may, however, be slower in those with diabetes, and pre-existing diabetic neuropathy may negatively influence surgical outcomes.

## Figures and Tables

**Table 1:** Risk for CTS in diabetes related to sex.

OR (95% CI)	Men with Diabetes		Women with Diabetes	
	1.99 (1.81–2.19)		2.63 (2.42–2.86)	
	T1D	T2D	T1D	T2D
Prevalence	6.8%	5.0%	13.5%	10.1%
Incidence rate/ 10,000 person-years	58.1	31.6	95.5	52.1

References: [9,12]. CI: confidence interval, OR: odds ratio, T1D: type 1 diabetes, T2D: type 2 diabetes.

## References

[1] Latinovic R., Gulliford M.C., Hughes R.A. (2006). Incidence of common compressive neuropathies in primary care. J. Neurol. Neurosurg. Psychiatry.

[2] Zimmerman M., Hall E., Carlsson K.S., Nyman E., Dahlin L.B. (2021). Socioeconomic factors predicting outcome in surgically treated carpal tunnel syndrome: A national registry-based study. Sci. Rep..

[3] Zimmerman M., Nyman E., Steen Carlsson K., Dahlin L.B. (2020). Socioeconomic Factors in Patients with Ulnar Nerve Compression at the Elbow: A National Registry-Based Study. BioMed Res. Int..

[4] Atroshi I., Gummesson C., Johnsson R., Ornstein E., Ranstam J., Rosen I. (1999). Prevalence of carpal tunnel syndrome in a general population. JAMA.

[5] Atroshi I. (2011). Incidence of physician-diagnosed carpal tunnel syndrome in the general population. Arch. Intern. Med..

[6] Tadjerbashi K., Åkesson A., Atroshi I. (2019). Incidence of referred carpal tunnel syndrome and carpal tunnel release surgery in the general population: Increase over time and regional variations. J. Orthop. Surg..

[7] Nordstrom D.L., DeStefano F., Vierkant R.A., Layde P.M. (1998). Incidence of diagnosed carpal tunnel syndrome in a general population. Epidemiology.

[8] Bland J.D., Rudolfer S.M. (2003). Clinical surveillance of carpal tunnel syndrome in two areas of the United Kingdom, 1991–2001. J. Neurol. Neurosurg. Psychiatry.

[9] Wiberg A., Ng M., Schmid A.B., Smillie R.W., Baskozos G., Holmes M.V., Künnapuu K., Mägi R., Bennett D.L., Furniss D. (2019). A genome-wide association analysis identifies 16 novel susceptibility loci for carpal tunnel syndrome. Nat. Commun..

[10] Renard E., Jacques D., Chammas M., Poirier J.L., Bonifacj C., Jaffiol C., Simon L., Allieu Y. (1994). Increased prevalence of soft tissue hand lesions in type 1 and type 2 diabetes mellitus: Various entities and associated significance. Diabete Metab..

[11] Papanas N., Maltezos E. (2010). The diabetic hand: A forgotten complication?. J. Diabetes Its Complicat..

[12] Rydberg M., Zimmerman M., Gottsäter A., Svensson A., Eeg-Olofsson K., Dahlin L.B. (2022). The Diabetic Hand-prevalence and incidence of diabetic hand problems using data from 1.1 million inhabitants in southern Sweden. BMJ Open Diabetes Res. Care.

[13] Rota E., Morelli N. (2016). Entrapment neuropathies in diabetes mellitus. World J. Diabetes.

[14] Rydberg M., Zimmerman M., Gottsäter A., Nilsson P.M., Melander O., Dahlin L.B. (2020). Diabetes mellitus as a risk factor for compression neuropathy: A longitudinal cohort study from southern Sweden. BMJ Open Diabetes Res. Care.

[15] Upton A.R., McComas A.J. (1973). The double crush in nerve entrapment syndromes. Lancet.

[16] Thomsen N.O., Mojaddidi M., Malik R.A., Dahlin L.B. (2009). Reduced myelinated nerve fibre and endoneurial capillary densities in the forearm of diabetic and non-diabetic patients with carpal tunnel syndrome. Acta Neuropathol..

[17] Dahlin L., Sanden H., Dahlin E., Zimmerman M., Thomsen N., Bjorkman A. (2014). Low myelinated nerve-fibre density may lead to symptoms associated with nerve entrapment in vibration-induced neuropathy. J. Occup. Med. Toxicol..

[18] Pop-Busui R., Boulton A.J., Feldman E.L., Bril V., Freeman R., Malik R.A., Sosenko J.M., Ziegler D. (2017). Diabetic Neuropathy: A Position Statement by the American Diabetes Association. Diabetes Care.

[19] Factors in development of diabetic neuropathy (1988). Baseline analysis of neuropathy in feasibility phase of Diabetes Control and Complications Trial (DCCT). The DCCT Research Group. Diabetes.

[20] Salvotelli L., Stoico V., Perrone F., Cacciatori V., Negri C., Brangani C., Pichiri I., Targher G., Bonora E., Zoppini G. (2015). Prevalence of neuropathy in type 2 diabetic patients and its association with other diabetes complications: The Verona Diabetic Foot Screening Program. J. Diabetes Its Complicat..

[21] Feldman E.L., Callaghan B.C., Pop-Busui R., Zochodne D.W., Wright D.E., Bennett D.L., Bril V., Russell J.W., Viswanathan V. (2019). Diabetic neuropathy. Nat. Rev. Dis. Primers.

[22] Partanen J., Niskanen L., Lehtinen J., Mervaala E., Siitonen O., Uusitupa M. (1995). Natural history of peripheral neuropathy in patients with non-insulin-dependent diabetes mellitus. N. Engl. J. Med..

[23] Aaberg M.L., Burch D.M., Hud Z.R., Zacharias M.P. (2008). Gender differences in the onset of diabetic neuropathy. J. Diabetes Its Complicat..

[24] Brownlee M. (2005). The pathobiology of diabetic complications: A unifying mechanism. Diabetes.

[25] Albers J.W., Pop-Busui R. (2014). Diabetic Neuropathy: Mechanisms, Emerging Treatments, and Subtypes. Curr. Neurol. Neurosci. Rep..

[26] Zimmerman M. (2018). The Diabetic Nerve. Studies on Outcome after Open Carpal Tunnel Release and the Development of Autonomic Neuropathy. Ph.D. Dissertation.

[27] Baptista F.I., Pinheiro H., Gomes C.A., Ambrósio A.F. (2019). Impairment of Axonal Transport in Diabetes: Focus on the Putative Mechanisms Underlying Peripheral and Central Neuropathies. Mol. Neurobiol..

[28] Medori R., Autilio-Gambetti L., Jenich H., Gambetti P. (1988). Changes in axon size and slow axonal transport are related in experimental diabetic neuropathy. Neurology.

[29] Mohseni S., Badii M., Kylhammar A., Thomsen N.O.B., Eriksson K.F., Malik R.A., Rosen I., Dahlin L.B. (2017). Longitudinal study of neuropathy, microangiopathy, and autophagy in sural nerve: Implications for diabetic neuropathy. Brain Behav..

[30] Callaghan B.C., Cheng H., Stables C.L., Smith A.L., Feldman E.L. (2012). Diabetic neuropathy: Clinical manifestations and current treatments. Lancet Neurol..

[31] Sima A.A.F., Zhang W., Grunberger G. (2004). Type 1 Diabetic Neuropathy and C-peptide. Exp. Diabesity Res..

[32] Nathan D.M. (2014). The diabetes control and complications trial/epidemiology of diabetes interventions and complications study at 30 years: Overview. Diabetes Care.

[33] Callaghan B.C., Little A.A., Feldman E.L., Hughes R.A. (2012). Enhanced glucose control for preventing and treating diabetic neuropathy. Cochrane Database Syst. Rev..

[34] Callaghan B., Feldman E. (2013). The metabolic syndrome and neuropathy: Therapeutic challenges and opportunities. Ann. Neurol..

[35] Callaghan B.C., Gallagher G., Fridman V., Feldman E.L. (2020). Diabetic neuropathy: What does the future hold?. Diabetologia.

[36] Eid S., Sas K.M., Abcouwer S.F., Feldman E.L., Gardner T.W., Pennathur S., Fort P.E. (2019). New insights into the mechanisms of diabetic complications: Role of lipids and lipid metabolism. Diabetologia.

[37] Ohkubo Y., Kishikawa H., Araki E., Miyata T., Isami S., Motoyoshi S., Kojima Y., Furuyoshi N., Shichiri M. (1995). Intensive insulin therapy prevents the progression of diabetic microvascular complications in Japanese patients with non-insulin-dependent diabetes mellitus: A randomized prospective 6-year study. Diabetes Res. Clin. Pract..

[38] (1998). Intensive blood-glucose control with sulphonylureas or insulin compared with conventional treatment and risk of complications in patients with type 2 diabetes (UKPDS 33). UK Prospective Diabetes Study (UKPDS) Group. Lancet.

[39] Osman A.A., Dahlin L.B., Thomsen N.O., Mohseni S. (2015). Autophagy in the posterior interosseous nerve of patients with type 1 and type 2 diabetes mellitus: An ultrastructural study. Diabetologia.

[40] Sunderland S. (1978). Nerves and Nerve Injuries.

[41] Boron W.F.B., Emile L. (2009). Medical Physiology.

[42] King R., Vallat J.-M., Weis J. (2014). Peripheral Nerve Disorders.

[43] Dahlin L.B., Shyu B.C., Danielsen N., Andersson S.A. (1989). Effects of nerve compression or ischaemia on conduction properties of myelinated and non-myelinated nerve fibres. An experimental study in the rabbit common peroneal nerve. Acta Physiol. Scand..

[44] Sleigh J.N., Rossor A.M., Fellows A.D., Tosolini A.P., Schiavo G. (2019). Axonal transport and neurological disease. Nat. Rev. Neurol..

[45] Dahlin L.B., Meiri K.F., McLean W.G., Rydevik B., Sjostrand J. (1986). Effects of nerve compression on fast axonal transport in streptozotocin-induced diabetes mellitus. An experimental study in the sciatic nerve of rats. Diabetologia.

[46] Snedeker J.G., Gautieri A. (2014). The role of collagen crosslinks in ageing and diabetes-the good, the bad, and the ugly. Muscles Ligaments Tendons J..

[47] Samii A., Unger J., Lange W. (1999). Vascular endothelial growth factor expression in peripheral nerves and dorsal root ganglia in diabetic neuropathy in rats. Neurosci. Lett..

[48] Lundborg G., Myers R., Powell H. (1983). Nerve compression injury and increased endoneurial fluid pressure: A “miniature compartment syndrome”. J. Neurol. Neurosurg. Psychiatry.

[49] Lakshminarayanan K., Shah R. (2021). Median nerve and carpal arch morphology changes in women with type 2 diabetes: A case–control study. J. Ultrasound.

[50] Mojaddidi M.A., Ahmed M.S., Ali R., Jeziorska M., Al-Sunni A., Thomsen N.O., Dahlin L.B., Malik R.A. (2014). Molecular and pathological studies in the posterior interosseous nerve of diabetic and non-diabetic patients with carpal tunnel syndrome. Diabetologia.

[51] Strömberg T., Dahlin L.B., Brun A., Lundborg G. (1997). Structural nerve changes at wrist level in workers exposed to vibration. Occup. Environ. Med..

[52] Vinik A., Mehrabyan A., Colen L., Boulton A. (2004). Focal entrapment neuropathies in diabetes. Diabetes Care.

[53] Mackinnon S.E. (2002). Pathophysiology of nerve compression. Hand Clin..

[54] Dahlin L.B. (1991). Aspects on pathophysiology of nerve entrapments and nerve compression injuries. Neurosurg. Clin. N. Am..

[55] Gupta R., Rowshan K., Chao T., Mozaffar T., Steward O. (2004). Chronic nerve compression induces local demyelination and remyelination in a rat model of carpal tunnel syndrome. Exp. Neurol..

[56] Aboonq M.S. (2015). Pathophysiology of carpal tunnel syndrome. Neurosciences.

[57] Lundborg G., Dahlin L.B. (1996). Anatomy, function, and pathophysiology of peripheral nerves and nerve compression. Hand Clin..

[58] Tapadia M., Mozaffar T., Gupta R. (2010). Compressive Neuropathies of the Upper Extremity: Pathophysiology, Classification, Electrodiagnostic Findings. J. Hand Surg..

[59] Thomsen N.O.B., Rosén I., Dahlin L.B. (2010). Neurophysiologic recovery after carpal tunnel release in diabetic patients. Clin. Neurophysiol..

[60] Thomsen N.O.B., Andersson G.S., Bjork J., Dahlin L.B. (2017). Neurophysiological recovery 5 years after carpal tunnel release in patients with diabetes. Muscle Nerve.

[61] Zhang D., Collins J., Blazar P., Earp B.E. (2020). Factors Associated With Advanced Presentation for Carpal Tunnel Release. J. Hand Surg. Am..

[62] Mackinnon S.E., Dellon A.L., Hudson A.R., Hunter D.A. (1986). Chronic human nerve compression—A histological assessment. Neuropathol. Appl. Neurobiol..

[63] Wiberg A., Smillie R.W., Dupré S., Schmid A.B., Bennett D.L., Furniss D. (2021). Replication of epidemiological associations of carpal tunnel syndrome in a UK population-based cohort of over 400,000 people. J. Plast. Reconstr. Aesthet. Surg..

[64] Pourmemari M.H., Shiri R. (2016). Diabetes as a risk factor for carpal tunnel syndrome: A systematic review and meta-analysis. Diabet. Med..

[65] Padua L., Padua R., Aprile, Tonali P. (1999). Italian multicentre study of carpal tunnel syndrome. Differences in the clinical and neurophysiological features between male and female patients. J. Hand Surg. Br..

[66] Caliandro P., Torre L.G., Padua R., Giannini F., Padua L. (2016). Treatment for ulnar neuropathy at the elbow. Cochrane Database Syst. Rev..

[67] Ennis S.L., Galea M.P., O’Neal D.N., Dodson M.J. (2016). Peripheral neuropathy in the hands of people with diabetes mellitus. Diabetes Res. Clin. Pract..

[68] Thomsen N.O., Englund E., Thrainsdottir S., Rosen I., Dahlin L.B. (2009). Intraepidermal nerve fibre density at wrist level in diabetic and non-diabetic patients. Diabet. Med..

[69] Yagci I., Gunduz O.H., Sancak S., Agirman M., Mesci E., Akyuz G. (2010). Comparative electrophysiological techniques in the diagnosis of carpal tunnel syndrome in patients with diabetic polyneuropathy. Diabetes Res. Clin. Pract..

[70] Dahlin E., Zimmerman M., Bjorkman A., Thomsen N.O., Andersson G.S., Dahlin L.B. (2016). Impact of smoking and preoperative electrophysiology on outcome after open carpal tunnel release. J. Plast. Surg. Hand Surg..

[71] Osiak K., Mazurek A., Pękala P., Koziej M., Walocha J.A., Pasternak A. (2021). Electrodiagnostic Studies in the Surgical Treatment of Carpal Tunnel Syndrome-A Systematic Review. J. Clin. Med..

[72] Perkins B.A., Olaleye D., Bril V. (2002). Carpal tunnel syndrome in patients with diabetic polyneuropathy. Diabetes Care.

[73] Dyck P.J., Kratz K.M., Karnes J.L., Litchy W.J., Klein R., Pach J.M., Wilson D.M., O’Brien P.C., Melton L.J., Service F.J. (1993). The prevalence by staged severity of various types of diabetic neuropathy, retinopathy, and nephropathy in a population-based cohort: The Rochester Diabetic Neuropathy Study. Neurology.

[74] Gerritsen A.A., de Vet H.C., Scholten R.J., Bertelsmann F.W., de Krom M.C., Bouter L.M. (2002). Splinting vs surgery in the treatment of carpal tunnel syndrome: A randomized controlled trial. JAMA.

[75] Hui A.C., Wong S., Leung C.H., Tong P., Mok V., Poon D., Li-Tsang C.W., Wong L.K., Boet R. (2005). A randomized controlled trial of surgery vs steroid injection for carpal tunnel syndrome. Neurology.

[76] Jarvik J.G., Comstock B.A., Kliot M., Turner J.A., Chan L., Heagerty P.J., Hollingworth W., Kerrigan C.L., Deyo R.A. (2009). Surgery versus non-surgical therapy for carpal tunnel syndrome: A randomised parallel-group trial. Lancet.

[77] Hulkkonen S., Lampainen K., Auvinen J., Miettunen J., Karppinen J., Ryhänen J. (2020). Incidence and operations of median, ulnar and radial entrapment neuropathies in Finland: A nationwide register study. J. Hand Surg. Eur. Vol..

[78] Haupt W.F., Wintzer G., Schop A., Lottgen J., Pawlik G. (1993). Long-term results of carpal tunnel decompression. Assessment of 60 cases. J. Hand Surg. Br..

[79] Ozkul Y., Sabuncu T., Kocabey Y., Nazligul Y. (2002). Outcomes of carpal tunnel release in diabetic and non-diabetic patients. Acta Neurol. Scand..

[80] Shin J., Kim Y.W., Lee S.C., Yang S.N., Chang J.S., Yoon S.Y. (2021). Effects of diabetes mellitus on the rate of carpal tunnel release in patients with carpal tunnel syndrome. Sci. Rep..

[81] Thomsen N.O.B., Cederlund R., Rosén I., Björk J., Dahlin L.B. (2009). Clinical Outcomes of Surgical Release Among Diabetic Patients With Carpal Tunnel Syndrome: Prospective Follow-Up With Matched Controls. J. Hand Surg..

[82] Thomsen N.O., Cederlund R.I., Andersson G.S., Rosen I., Bjork J., Dahlin L.B. (2014). Carpal tunnel release in patients with diabetes: A 5-year follow-up with matched controls. J. Hand Surg. Am..

[83] Jenkins P.J., Duckworth A.D., Watts A.C., McEachan J.E. (2012). The outcome of carpal tunnel decompression in patients with diabetes mellitus. J. Bone Jt. Surg. Br. Vol..

[84] Isik C., Uslu M., Inanmaz M.E., Karabekmez F.E., Kose K.C. (2013). The effects of diabetes on symptoms of carpal tunnel syndrome treated with mini-open surgery. Acta Orthop. Belg..

[85] Zyluk A., Puchalski P. (2013). A comparison of outcomes of carpal tunnel release in diabetic and non-diabetic patients. J. Hand Surg. Eur. Vol..

[86] Cagle P.J., Reams M., Agel J., Bohn D. (2014). An outcomes protocol for carpal tunnel release: A comparison of outcomes in patients with and without medical comorbidities. J. Hand Surg. Am..

[87] Gulabi D., Cecen G., Guclu B., Cecen A. (2014). Carpal tunnel release in patients with diabetes result in poorer outcome in long-term study. Eur. J. Orthop. Surg. Traumatol. Orthop. Traumatol..

[88] Watchmaker J.D., Watchmaker G.P. (2017). Independent Variables Affecting Outcome of Carpal Tunnel Release Surgery. Hand.

[89] Zimmerman M., Dahlin E., Thomsen N.O., Andersson G.S., Bjorkman A., Dahlin L.B. (2016). Outcome after carpal tunnel release: Impact of factors related to metabolic syndrome. J. Plast. Surg. Hand Surg..

[90] Zimmerman M., Eeg-Olofsson K., Svensson A., Astrom M., Arner M., Dahlin L. (2019). Open carpal tunnel release and diabetes: A retrospective study using PROMs and national quality registries. BMJ Open.

[91] Moradi A., Sadr A., Ebrahimzadeh M.H., Hassankhani G.G., Mehrad-Majd H. (2020). Does diabetes mellitus change the carpal tunnel release outcomes? Evidence from a systematic review and meta-analysis. J. Hand Ther..

[92] de Rijk M.C., Vermeij F.H., Suntjens M., van Doorn P.A. (2007). Does a carpal tunnel syndrome predict an underlying disease?. J. Neurol. Neurosurg. Psychiatry.

[93] Werner B.C., Teran V.A., Deal D.N. (2018). Patient-Related Risk Factors for Infection Following Open Carpal Tunnel Release: An Analysis of Over 450,000 Medicare Patients. J. Hand Surg. Am..

[94] Harness N.G., Inacio M.C., Pfeil F.F., Paxton L.W. (2010). Rate of Infection After Carpal Tunnel Release Surgery and Effect of Antibiotic Prophylaxis. J. Hand Surg..

[95] Werner B.C., Teran V.A., Cancienne J., Deal D.N. (2019). The Association of Perioperative Glycemic Control With Postoperative Surgical Site Infection Following Open Carpal Tunnel Release in Patients With Diabetes. Hand.

[96] Collins P.S., Apel P.J., Truong A.Y., Zarei M., Lozano A.J., Capito A.E. (2022). The Utility of Preoperative HbA1c as a Standardized Protocol in Elective Carpal Tunnel Release: A Retrospective Review of Clinical Outcomes. Hand.

[97] Al-Qattan M.M., Manktelow R.T., Bowen C.V. (1994). Outcome of carpal tunnel release in diabetic patients. J. Hand Surg. Br..

[98] Choi S.J., Ahn D.S. (1998). Correlation of clinical history and electrodiagnostic abnormalities with outcome after surgery for carpal tunnel syndrome. Plast. Reconstr. Surg..

[99] Mondelli M., Padua L., Reale F., Signorini A.M., Romano C. (2004). Outcome of surgical release among diabetics with carpal tunnel syndrome. Arch. Phys. Med. Rehabil..

[100] Ebrahimzadeh M.H., Mashhadinejad H., Moradi A., Kachooei A.R. (2013). Carpal tunnel release in diabetic and non-diabetic patients. Arch. Bone Jt. Surg..

[101] Yucel H. (2015). Factors affecting symptoms and functionality of patients with carpal tunnel syndrome: A retrospective study. J. Phys. Ther. Sci..

[102] Zhang D., Blazar P., Earp B.E. (2018). Rates of Complications and Secondary Surgeries of Mini-Open Carpal Tunnel Release. Hand.

[103] Dellon A.L. (2014). Susceptibility of nerve in diabetes to compression: Implications for pain treatment. Plast. Reconstr. Surg..

[104] Sarmiento S., Pierre J.A., Dellon A.L., Frick K.D. (2019). Tibial nerve decompression for the prevention of the diabetic foot: A cost-utility analysis using Markov model simulations. BMJ Open.

[105] Dellon A.L., Muse V.L., Nickerson D.S., Akre T., Anderson S.R., Barrett S.L., Biddinger K.R., Bregman P.J., Bullard B.P., Dauphinee D.M. (2012). Prevention of ulceration, amputation, and reduction of hospitalization: Outcomes of a prospective multicenter trial of tibial neurolysis in patients with diabetic neuropathy. J. Reconstr. Microsurg..

[106] Cornblath D.R., Vinik A., Feldman E., Freeman R., Boulton A.J. (2007). Surgical decompression for diabetic sensorimotor polyneuropathy. Diabetes Care.

[107] Nickerson D.S. (2017). Nerve decompression and neuropathy complications in diabetes: Are attitudes discordant with evidence?. Diabet. Foot Ankle.

[108] Chaudhry V., Stevens J.C., Kincaid J., So Y.T. (2006). Practice Advisory: Utility of surgical decompression for treatment of diabetic neuropathy: Report of the Therapeutics and Technology Assessment Subcommittee of the American Academy of Neurology. Neurology.

[109] Rinkel W.D., Fakkel T.M., Castro Cabezas M., Birnie E., Coert J.H. (2020). (Cost-)effectiveness of lower extremity nerve decompression surgery in subjects with diabetes: The DeCompression (DECO) trial-study protocol for a randomised controlled trial. BMJ Open.

[110] Dahlin L.B., Rix K.R., Dahl V.A., Dahl A.B., Jensen J.N., Cloetens P., Pacureanu A., Mohseni S., Thomsen N.O.B., Bech M. (2020). Three-dimensional architecture of human diabetic peripheral nerves revealed by X-ray phase contrast holographic nanotomography. Sci. Rep..

[111] Ising E., Åhrman E., Thomsen N.O.B., Eriksson K.F., Malmström J., Dahlin L.B. (2021). Quantitative proteomic analysis of human peripheral nerves from subjects with type 2 diabetes. Diabet. Med..

